# The Homogentisate and Homoprotocatechuate Central Pathways Are Involved in 3- and 4-Hydroxyphenylacetate Degradation by *Burkholderia xenovorans* LB400

**DOI:** 10.1371/journal.pone.0017583

**Published:** 2011-03-10

**Authors:** Valentina Méndez, Loreine Agulló, Myriam González, Michael Seeger

**Affiliations:** Laboratorio de Microbiología Molecular y Biotecnología Ambiental, Departamento de Química and Center for Nanotechnology and Systems Biology, Universidad Técnica Federico Santa María, Valparaíso, Chile; University of Groningen, Netherlands

## Abstract

**Background:**

Genome characterization of the model PCB-degrading bacterium *Burkholderia xenovorans* LB400 revealed the presence of eleven central pathways for aromatic compounds degradation, among them, the homogentisate and the homoprotocatechuate pathways. However, the functionality of these central pathways in strain LB400 has not been assessed and related peripheral pathways has not been described.

**Methodology/Principal Findings:**

The aims of this study were to determine the functionality of the homogentisate and homoprotocatechuate central pathways in *B. xenovorans* LB400 and to establish their role in 3-hydroxyphenylacetate (3-HPA) and 4-hydroxyphenylacetate (4-HPA) catabolism. Strain LB400 was able to grow using 3-HPA and 4-HPA as sole carbon source. A genomic search in LB400 suggested the presence of *mhaAB* and *hpaBC* genes clusters encoding proteins of the 3-hydroxyphenylacetate and 4-hydroxyphenylacetate peripheral pathways. LB400 cells grown with 3-HPA and 4-HPA degraded homogentisate and homoprotocatechuate and showed homogentisate 1,2-dioxygenase and homoprotocatechuate 2,3-dioxygenase activities. Transcriptional analyses by RT-PCR showed the expression of two chromosomally-encoded homogentisate dioxygenases (BxeA2725 and BxeA3900) and the *hpaD* gene encoding the homoprotocatechuate 2,3-dioxygenase during 3-HPA and 4-HPA degradation. The proteome analyses by two-dimensional polyacrilamide gel electrophoresis of *B. xenovorans* LB400 grown in 3-HPA and 4-HPA showed the induction of fumarylacetoacetate hydrolase HmgB (BxeA3899).

**Conclusions/Significance:**

This study revealed that strain LB400 used both homogentisate and homoprotocatechuate ring-cleavage pathways for 3- hydroxyphenylacetate and 4-hydroxyphenylacetate catabolism and that these four catabolic routes are functional, confirming the metabolic versatility of *B. xenovorans* LB400.

## Introduction

Aromatic compounds are widely distributed in ecosystems mainly released from plant materials and by anthropogenic activities. Bacteria have evolved to metabolize diverse aromatic compounds including environmental pollutants, using these compounds as carbon and energy source. A broad range of aromatic compounds is degraded by bacteria through peripheral pathways that funnel into few central pathways [1]–[3]. In peripheral pathways, activation of the aromatic ring is commonly mediated by monooxygenases or dioxygenases that produce dihydroxylated intermediates. Central pathways involve the fission by a dioxygenase of the dihydroxylated aromatic metabolic intermediates at *ortho*- or *meta*-position and lead to the formation of Krebs cycle intermediates [1]–[4]. Bacterial metabolism of aromatic compounds has been extensively studied [3]–[11]. The metabolic reconstruction of aromatic compounds pathways in in model environmental bacteria has been achieved by genome sequence analyses [3], [12]–[14]. However, further studies are needed to establish gene function and to elucidate functional aromatic metabolic pathways of bacteria.

*B. xenovorans* LB400 is a model bacterium for the degradation of PCBs and other aromatic compounds [3], [15], [16]. Strain LB400 has one of the largest bacterial genomes (9.73 Mbp), distributed in a major chromosome (C1), a minor chromosome (C2) and a megaplasmid (MP). Genome analyses of strain LB400 revealed the presence of genes encoding eleven central pathways and twenty peripheral pathways for aromatic compounds degradation [3]. The genes encoding the homogentisate and the homoprotocatechuate central pathways were identified in *B. xenovorans* LB400 genome [3]. The *hmgABC* and *hpaGEDFHI* gene clusters are located at C1 and C2, respectively. Additional *hmg* gene copies were identified within LB400 genome [3]. Both ring-cleavage pathways are used in aromatic amino acid metabolism in *Bacteria* and *Eucarya* [17]–[20]. The homogentisate pathway has been described as the central route for the degradation of L-tyrosine and L-phenylalanine in bacteria, fungi and mammals [19], [21], [22]. The homogentisate central pathway is encoded by the *hmgABC* operon in *Pseudomonas putida* strain U [19]. Homogentisate (HMG) cleavage is catalysed by the HMG 1,2-dioxygenase HmgA producing maleylacetoacetate (MA). MA is isomerized by a maleylacetoacetate isomerase (HmgC) into fumarylacetoacetate, which is further converted by a fumarylacetoacetate hydrolase (HmgB) into fumarate and acetoacetate [23]. The homoprotocatechuate pathway has been described as a central route for the catabolism of aromatic amino acids and related compounds in *Klebsiella pneumoniae, P. putida*, and *Escherichia coli* [2], [24]–[28]. The homoprotocatechuate pathway is encoded by the *hpaGEDFHI* gene cluster in *E. coli* strain W. Homoprotocatechuate (HPC) is *meta*-cleaved by a HPC 2,3-dioxygenase encoded by the *hpaD* gene. The product 5-carboxymethyl-2-hydroxymuconic semialdehyde (CHMS) is then converted to 5-carboxymethyl-2-hydroxy-muconic acid (CHM) and subsequently degraded by the *hpaF*, *hpaG*, *hpaH* and *hpaI* gene products into Krebs cycle intermediates [2], [17], [18], [28].

Degradation of phenylacetate hydroxylated-derivatives is channeled into the HMG or the HPC central pathways [17], [20], [29], [30]. Assimilation of 3-hydroxyphenylacetate (3-HPA) through the homogentisate pathway in *P. putida* U has been reported. The *mhaAB* genes encode a 3-HPA 6-hydroxylase that hydroxylates 3-HPA at C-6 producing HMG [19], [30]. In addition, *P. putida* strain U degrades 4-hydroxyphenylacetate (4-HPA) via the homoprotocatechuate pathway [27]. *E. coli* strains W, B and C are able to degrade both 3-HPA and 4-HPA via the homoprotocatechuate central route, which is encoded by the inducible *hpaGEDFHI* operon [2], [18], [24], [26]. The *hpaBC* gene cluster encodes a 4-HPA 3-hydroxylase involved in the hydroxylation of 4-HPA and 3-HPA to yield HPC [18]. The *hpaBC* and *hpaGEDFHI* gene clusters constitute a single operon in *E. coli* strain W [2], [18].

The aims of this study were to determine functional homogentisate and homoprotocatechuate central pathways in *B. xenovorans* strain LB400 and to establish their role in 3-HPA and 4-HPA peripheral pathways, which has not been described previously in this bacterium. This study showed that both homogentisate and homoprotocatechuate central pathways are involved in 3-HPA and 4-HPA degradation in strain LB400.

## Materials and Methods

### Chemicals

3-hydroxyphenylacetic acid (>99% purity), 4-hydroxyphenylacetic acid (98% purity) and 3,4-dihydroxyphenylacetic acid (homoprotocatechuate; 98% purity) and 2,5-dihydroxyphenylacetic acid (homogentisate; 98% purity) were obtained from Sigma-Aldrich (Saint Louis, MO, USA).

### Bacterial strain and culture conditions

*Burkholderia xenovorans* LB400 was cultivated at 30°C in mineral M9 medium with trace solution and glucose (5 mM) [31], 3-HPA (5 mM) or 4-HPA (5 mM) as the sole carbon and energy source. Growth was determined by measuring turbidity at 525 nm and by counting colony-forming units (CFU). Aliquots taken from cultures were diluted and plated on Luria-Bertani agar medium. CFU per milliliter values were calculated as the mean ± SD of at least three independent experiments.

### Resting cell assays

Resting cells (turbidity _525nm_ = 2.0) in 50 mM sodium phosphate buffer (pH 7.0) were incubated with HMG or HPC (0.03 mM) at 30°C. Aliquots of cell suspensions were taken at different incubation times and centrifuged (19,283 *g* for 2 min). Assays with boiled cells and without cells were used as controls. Cell-free supernatants were analyzed, using a Waters liquid chromatograph model 515 equipped with a UV detector and a RP-C18/Lichrospher 5-µm column (Supelco, Bellefonte, USA). The mobile phase contained 20% acetonitrile, 20% methanol, 60% water and 0.1% phosphoric acid. The flow rate was 0.5 mL min^-1^. HMG and HPC had retention times of 4.6 and 5.1 min, respectively. HMG and HPC were quantified using calibration curves with authentic standards. Resting cells experiments were performed in triplicate.

### Preparation of cell extracts

Cells harvested at exponential phase of growth (turbidity_525 nm_ = 0.40–0.45) were centrifuged (10,733 *g* for 10 min) at 4°C and washed with 50 mM sodium phosphate buffer (pH 7.0). Bacterial cells were disrupted using an ultrasonic cell disruptor Microson™ at 4°C. Lysate was clarified by centrifugation (19,300 g for 15 min) at 4°C. Protein concentration was determined using Qubit™ fluorometer (Invitrogen).

### Dioxygenase activity assays

HMG dioxygenase activity was determined spectrophotometrically by measuring the formation of MA at 330 nm as previously described [32]. The assay volume (1 ml) contained: 50 mM phosphate buffer (pH 7.0), 50 µM FeSO_4_, 2 mM ascorbate, crude extract (100 µg of protein) and 0.3 mM HMG. The reactions were carried out at 30°C and initiated by the addition of HMG. One enzyme milliunit corresponds to the transformation of 1 µmol of HMG to MA per min at 30°C under the conditions described above. HMG dioxygenase activity was calculated using the molar extinction coefficient of MA, 13,500 M^−1^ cm^−1^ [33]. HPC dioxygenase activity was measured by monitoring the formation of 5-carboxymethyl-2-hydroxymuconate semialdehyde (CHMS) at 380 nm [34]. LB400 cells cultivated using 3-HPA, 4-HPA or glucose were harvested at a turbidity_525 nm_ of 0.45, washed and concentrated. HPC dioxygenase activity of LB400 cell suspension (turbidity_525 nm_  = 2.0) was determined by incubation with 0.3 mM HPC at 30°C. At intervals, the A_380nm_ values of supernatants were measured.

### Two-dimensional polyacrylamide gel electrophoresis (2-DE)

2-DE gels with non-equilibrium pH gradient electrophoresis (pH 3–10) were performed as previously described [31], [35]. Cell cultures were grown using glucose, 3-HPA or 4-HPA (5 mM) as sole carbon source. Cells were harvested at exponential phase (turbidity_525nm_  = 0.40–0.45) by centrifugation, disrupted with a sonicator and concentrated in a speed-vac concentrator. Dried samples were resuspended in lysis buffer (9.5 M urea, 2% v/v IGEPAL CA-630, 2% ampholytes (1.6% ampholytes pH 5–7, 0.4% ampholytes pH 3–10, Bio-Rad) and 5% β-mercaptoethanol. NEPHGE gels were electrophoresed for 6.5 h at 400 V. The second dimension was performed in 11% polyacrilamide-SDS gels. Proteins were visualized with Coomassie brilliant blue R-250. The volume (Int × mm^2^) of the spots was analyzed, using Quantity One image analysis software (Bio-Rad Laboratories). To standardize the quantification of independent experiments, the ratio of the intensity of the protein to a reference protein was calculated.

### Protein sequencing and identification

To perform the mass spectrometric analysis, protein spots were recovered from gels and prepared as described [31]. For matrix-assisted laser desorption ionization-time of flight (MALDI-TOF) analysis, the peptide extracts were analysed with a MALDI-TOF UltraXex apparatus (Bruker, Bremen, Germany). The peptide fingerprints obtained by mass spectrometry MALDI-TOF were used for searches in the NCBI protein database with the Matrix science MASCOT search tool. The complete sequences of each protein were obtained and BLAST searches were executed with the FASTA tool for the identification and similarity data analyses.

### Isolation of total RNA and RT-PCR

Total RNA was isolated from LB400 cells in the stationary growth phase using an RNeasy mini kit (Qiagen, Hilden, Germany) according to the manufacturers' recommendations. DNase I treatment was carried out using the RNase-Free DNase Set (Qiagen, Hilden, Germany) to degrade any residual DNA. The RNA concentration was quantified using a Qubit™ fluorometer (Invitrogen, Carlsbad, CA, USA). Reverse transcription-PCR (RT-PCR) was carried out with sequence-specific primers design in this study for *hmgA1* (BxeA2725), *hmgA2* (BxeA3900) and *hpaD* (BxeB2031) genes by using SuperScript™ One-step RT-PCR with Platinum®*Taq* (Invitrogen, Carlsbad, CA, USA). The BxeA2725 gene (*hmgA1*) was amplified using the primers HmgA1-f (5′-ATTTGCGACCGAAACGCTGCC-3′) and HmgA1-r (5′-CGACGGGTTGAAGTGCTTCC-3′). Amplification of the BxeA3900 gene (*hmgA2*) was performed with specific primers HmgA2-f (5′-TACGCCGAACACTGTCAGTTCG-3′) and HmgA2-r (5′-GCCCCGAAATTCTCGCAGATGTA-3′). Reverse transcriptase reaction for *hpaD* mRNA was performed using the primers HpaD-f (5′-CTCGCGTTGGCAGCAAAAGTG-3′) and HpaD-r (5′-GCAGAGGCGTAACCGGAAAG-3′). Amplification of the 16S rRNA gene was used as control for DNA contamination using the primers 27f (5′-AGAGTTTGATCMTGGCTCAG-3′) and 1492r (5′-TACGGYTAC CTTGTTACGACTT-3′) [36].

## Results

### Analysis of *hmg* and *hpa* gene clusters in strain LB400

**Figure 1:**
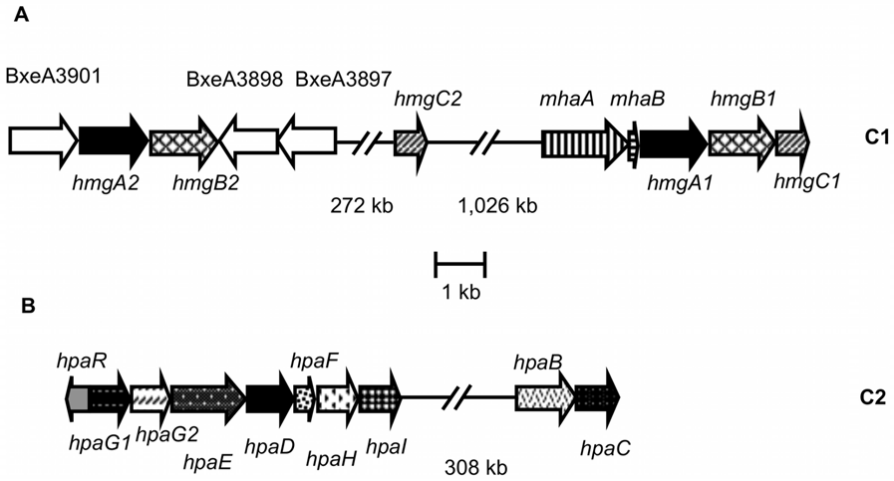
Organization of genes encoding the homogentisate and homoprotocatechuate central pathways and related peripheral pathways in *B. xenovorans* LB400. A, The *hmg* genes encoding the homogentisate central pathway, and *mha* genes encoding a 3-HPA peripheral pathway located in the major chromosome (C1). B, The *hpa* cluster encoding the homoprotocatechuate central pathway, and *hpaBC* genes encoding a 4-HPA peripheral pathway located in the minor chromosome (C2) of strain LB400. Genes encoding ring-cleavage dioxygenases are indicated with black arrows.

**Figure 2:**
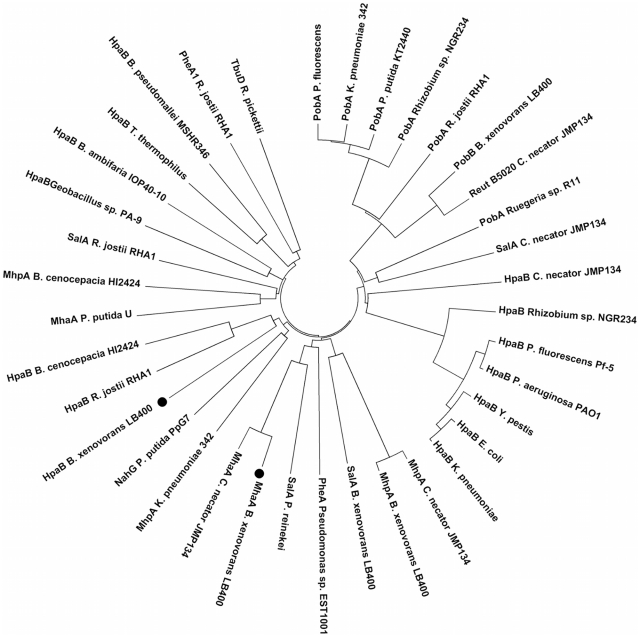
Phylogenetic tree showing the relatedness of FAD-dependent monooxygenases. The dendogram was constructed by the neighbor-joining method using MEGA 4.1 based on sequence alignments calculated by Clustal W. Sequences of deduced proteins from BxeA2727 and BxeB2308 genes from *B. xenovorans* L400 are highlighted (black circles). Proteins and their accession numbers are: HpaB (Bxe_B2308) *B. xenovorans* LB400 (YP_553029); HpaB *E. coli* (CAA82321); HpaB *K. pneumoniae,* (AAC37120); HpaB *T. thermophilus* (BAD70783); HpaB *Geobacillus sp.* PA-9 (AAT28189); HpaB *P. fluorescens* Pf-5 (YP_260461); HpaB *Y. pestis* (YP_651053); HpaB *Rhizobium sp.* NGR234, (YP_002824209); HpaB *P. aeruginosa* PAO1 (NP_252780); HpaB *R. jostii* RHA1, (YP_701753); HpaB *B. ambifaria* IOP40-10 (ZP_02892641); HpaB *B. pseudomallei* MSHR346 (ZP_04521853); HpaB *C. necator* JMP134 (YP_293474); HpaB *B. cenocepacia* HI2424 (YP_837331); MhaA (BxeA2727) *B. xenovorans* LB400, (ABE30237); MhaA *P. putida* U (AAY16572); MhaA *C. necator* JMP134 (AAZ64667); MhpA *K. pneumoniae* 342 (YP_002238037); MhpA *B. cenocepacia* HI2424 (YP_840344), MhpA *C. necator* JMP134 (YP_294496); MhpA *B. xenovorans* LB400 (YP_553009); PheA *Pseudomonas sp.* EST1001 (AAC64901); PheA1 *R. jostii* RHA1 (YP_702477); TbuD *R. pickettii* (AAA25992); PobB *B. xenovorans* LB400 (ABE30926); PobA *P. putida* KT2440 (AAN69138); PobA *P. fluorescens* (CAA48483); Reut_B5020 *C. necator* JMP134 (AAZ64368); PobA *Rhizobium sp.* NGR234 (YP_002823464); PobA *Ruegeria sp.* R11 (ZP_05090233); PobA *R. jostii* RHA1 (YP_702502); PobA *K. pneumoniae* 342 (YP_002240587); SalA *B. xenovorans* LB400 (ABE36893); NahG *P. putida* PpG7 (AAA25897); SalA *P. reinekei* (ABH07020); SalA *C. necator* JMP134 (YP_296720); SalA *R. jostii* RHA1 (YP_701838).

The *hmgABC* gene cluster (BxeA2725; BxeA2724 and BxeA2723; hereafter *hmgA1*, *hmgB1* and *hmgC1*, respectively) is located at C1 of strain LB400 genome. Copies of the *hmgA1* gene and *hmgB1* genes (BxeA3900 and BxeA3899, respectively) were identified in a 1,300 kb distant region from *hmgA1B1C1* cluster at C1 (hereafter *hmgA2* and *hmgB2*). A *hmgC* gene copy (BxeA4141; hereafter *hmgC2*) is located in a different region of C1 [3] (Fig. 1A). Sequence analyses of the chromosomal region flanking the *hmgA1B1C1* gene cluster in the strain LB400, revealed that the products of the open reading frames (ORFs) BxeA2727 and BxeA2726 shared 47% and 35% sequences identity with the MhaA and MhaB proteins from *P. putida* strain U, respectively [30] (Fig. 1A). In *P. putida* U the enzyme 3-HPA 6-hydroxylase (MhaAB) is a two- component FAD-dependent monooxygenase that hydroxylates 3-HPA to produce HMG (Fig. 2). The large component MhaA is a flavoprotein and the small component MhaB is a coupling protein [30]. In this study, the presence of the *mhaAB* genes located adjacent of *hmgA1B1C1* gene cluster in LB400 was revealed, suggesting a role of *mhaAB* genes in 3-HPA peripheral reaction leading to HMG.

The gene cluster *hpaG1G2EDFHI* encoding the homoprotocatechuate pathway is located at C2 in strain LB400 genome (BxeB2028, BxeB2029, BxeB2030, BxeB2031, BxeB2032, BxeB2033 and BxeB2034) (Fig. 1B). Downstream and adjacent to the *hpa* gene cluster, a divergent gene putatively encoding the HpaR repressor protein of the HPC catabolic pathway was identified (Fig. 1B) [3]. Genes encoding the 4-HPA catabolic pathway were not found in the neighborhood of *hpa* gene cluster in strain LB400. The degradation of 4-HPA in *E. coli* strain W involves enzymes encoded in the 4-HPA hydroxylase *hpaBC* operon and the *meta*-cleavage operon *hpaGEDFHI* [18]. The *hpaBC* cluster of strain W encodes a two-component protein responsible for 4-HPA hydroxylation to yield HPC [18], [29]. The BxeB2309 gene product of strain LB400 was identified as the 4-HPA 3-monooxygenase coupling protein HpaC located downstream to the *hpa* cluster (at 309 kb). The protein encoded by the BxeB2308 gene is highly related to the HpaB proteins from *B. cenocepacia* strain HI2424 and *R. jostii* RHA1 (Fig. 2). Based on its relatedness with FAD-dependent monooxygenases and its location next to a putative *hpaC* gene, the gene BxeB2308 may encode the main component of the closely related 4-HPA 3-monooxygenase (HpaB). However, further analyses are required to establish the 4-HPA 3-monooxygenase peripheral pathway encoded by BxeB2308 and BxeB2309 genes in LB400. The presence of genes putatively encoding peripheral reactions leading the homogentisate and homoprotocatechuate central pathways suggested functional HPA peripheral pathways leading to these ring-cleavage pathways in strain LB400.

### Strain LB400 growth on HPAs

Sequence analyses of strain LB400 genome revealed the presence of putative genes encoding 3-HPA and 4-HPA peripheral pathways. To this end, the growth of strain LB400 on 3-HPA and 4-HPA as sole carbon source was studied. Strain LB400 was able to grow using 3-HPA and 4-HPA as sole carbon and energy source reaching a high biomass (data not shown). Strain LB400 attained at stationary phase (25 h), slightly higher biomass using 4-HPA (3.21× FU 10^7^ mL^−1^) than using 3-HPA (1.28 1× CFU 10^7^ mL^−1^) and glucose (2.09× CFU 10^7^ mL^−1^) as sole carbon source. The growth on HPAs indicate that strain LB400 possess functional 3-HPA and 4-HPA catabolic pathways.

### HMG and HPC degradation by strain LB400

**Figure 3:**
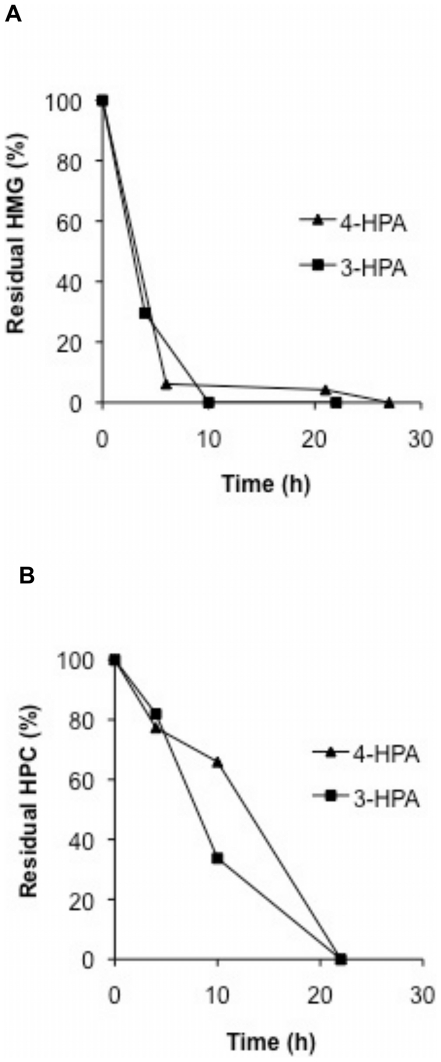
Degradation of HMG and HPC by resting cells of *B. xenovorans* LB400. LB400 cells were grown in 3-HPA (squares) or 4-HPA (triangles) as sole carbon source and incubated with HMG (0.03 mM) (A) or HPC (0.03 mM) (B). Control assays with boiled cells showed no degradation of HMG and HPC (data not shown). Each point is an average of results from at least three independent assays.

In order to determine the functionality of the homogentisate and homoprotocatechuate central pathways, the degradation of these compounds was analysed. HMG and HPC degradation by strain LB400 were studied using resting cell assays. LB400 cells grown either in 3-HPA or 4-HPA were able to degrade HMG and HPC compounds. Cells grown in 3-HPA and 4-HPA degraded after 10 h of incubation 100% and >95% of HMG, respectively (Fig. 3A). It is worth noting that, during HMG degradation assays, a brown colored medium was only observed in control assays with boiled cells. In contrast, no pigmented medium was observed in assays with metabolically active cells. The brown pigmentation of the medium has been reported in bacteria and fungi that lack the homogentisate pathway, and thus HMG accumulation and its chemical oxidation were observed [19], [20], [37]. LB400 cells grown in 3-HPA and 4-HPA degraded after 22 h almost completely HPC (Fig. 3B). A slightly faster degradation of HPC was observed with 3-HPA-grown cells than with 4-HPA-grown cells. These results showed that strain LB400 degraded HMG and HPC, indicating that the corresponding ring-cleavage pathways are active in 3-HPA- and 4-HPA-grown cells and are involved in their degradation.

### HMG and HPC ring-cleavage activities

**Figure 4:**
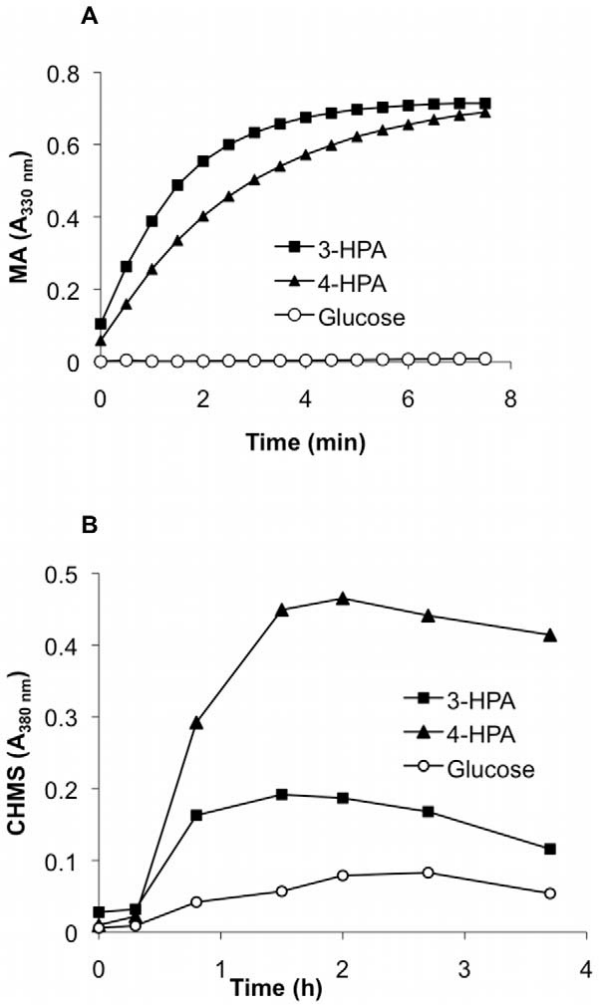
Homogentisate and homoprotocatechuate ring-cleavage dioxygenase activities of *B. xenovorans* LB400. Cells were grown in minimal medium using 3-HPA (squares), 4-HPA (triangles) and glucose (empty circles) as sole carbon source. A, HMG dioxygenase activity measured by maleylacetoacetate (MA) formation in crude extracts; B, HPC dioxygenase activity measured 5-carboxymethyl-2-hydroxy-muconic semialdehyde (CHMS) product formation in resting cells of strain LB400.

To further determine functional homogentisate and homoprotocatechuate central pathways, the enzymatic activities of HMG dioxygenase and HPC dioxygenase were determined. HMG dioxygenase activity was measured in crude extracts of LB400 cells cultured in 3-HPA, 4-HPA and glucose. A high HMG dioxygenase activity was observed during growth on 3-HPA (82 mU/mg protein) and 4-HPA (75 mU/mg protein) (Fig. 4A). HMG dioxygenase activity was slightly higher on 3-HPA-grown cells than on 4-HPA-grown cells, whereas no activity was observed in glucose-grown cells. HPC dioxygenase activity was measured by monitoring the formation of CHMS after whole cells incubation with HPC. Cells grown on 3-HPA and 4-HPA showed high HPC dioxygenase activity. A moderately higher HPC dioxygenase activity was observed in 4-HPA-grown cells than in 3-HPA-grown cells. In contrast, a low HPC dioxygenase activity was measured in glucose grown cells (Fig. 4B). These results indicate an induction of the key enzymes of the homogentisate and homprotocatechuate central pathways during growth of strain LB400 on 3-HPA and 4-HPA.

### *hmgA* and *hpaD* expression analyses by RT-PCR

**Figure 5:**
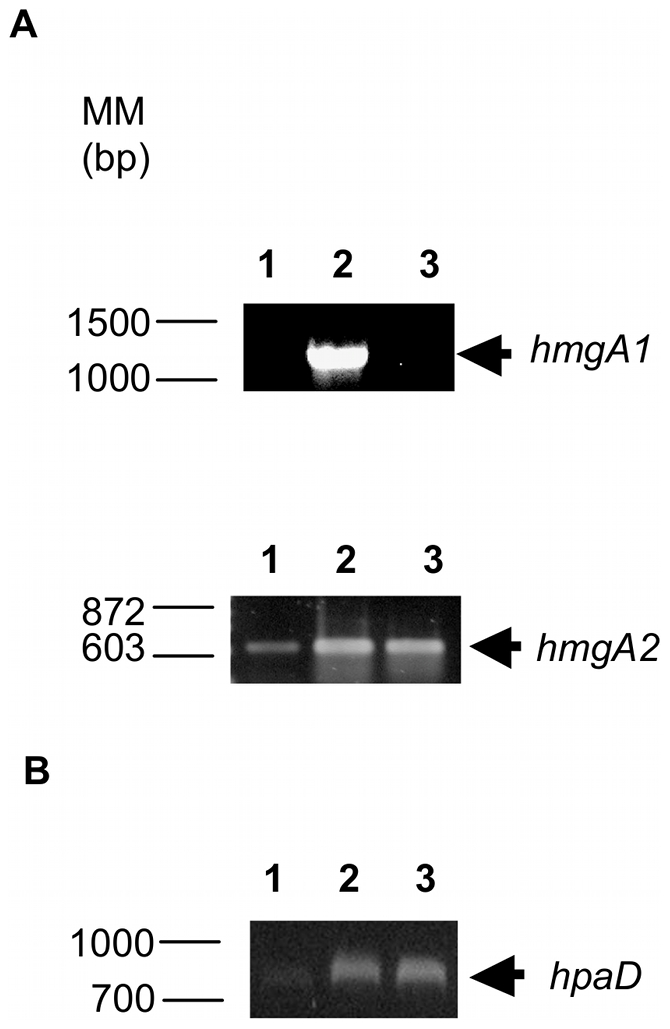
Expression of *hmgA* and *hpaD* genes during growth on HPAs. LB400 cells were grown on glucose (lanes 1) 3-HPA (lanes 2) and 4-HPA (lanes 3) as sole carbon source. A, Expression of *hmgA1* (BxeA2725); B, expression of *hmgA2* (BxeA3900); C, expression of *hpaD*. RT-PCR assays were performed using RNA from LB400 cells collected at early exponential growth phase.

Further analyses were conducted in order to determine the role of the homogentisate and homoprotocatechuate pathways in 3-HPA and 4-HPA catabolism. The expression of *hmgA* and *hpaD* genes encoding the key enzymes HMG and HPC dioxygenases was analyzed during exponential growth of strain LB400 in 3-HPA and 4-HPA. Additionally, transcriptional analyses of two gene copies encoding HMG 1,2-dioxygenase were performed using specific primers for *hmgA1* and *hmgA2* genes. Expression of the *hmgA1* gene was observed at early exponential growth phase of strain LB400 in 3-HPA, whereas no expression was detected during growth on 4-HPA or glucose (Fig. 5A). This indicates that *hmgA1* gene is up regulated by 3-HPA in strain LB400. On the other hand, expression of the *hmgA2* gene was detected in 3-HPA, 4-HPA and glucose grown-cells. These data also suggests lower *hmgA2* transcription in glucose-grown cells (Fig. 5A).

Expression analysis of *hpaD* gene encoding HPC 2,3-dioxygenase was performed. The *hpaD* transcripts were detected at early exponential phase of strain LB400 during growth on 3-HPA, 4-HPA and glucose as sole carbon source (Fig. 5B). Higher *hpaD* expression during growth on 3-HPA and 4-HPA was observed.

These results indicate an expression of the dioxygenase-encoding genes during 3-HPA and 4-HPA catabolism in strain LB400. Furthermore, these results indicate that during 3-HPA degradation, two HMG 1,2-dioxygenase encoding *hmgA* genes are expressed.

### Proteomic analyses during 3-HPA and 4-HPA degradation

**Figure 6:**
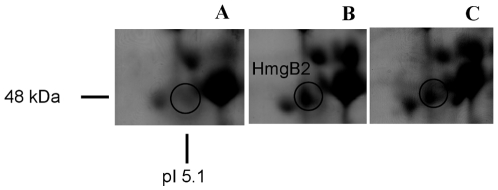
Induction of fumarylacetoacetate hydrolase HmgB in *B. xenovorans* LB400 during growth on HPAs. Cells were grown on glucose 5 mM (A), 3-HPA 5 mM (B) and 4HPA 5 mM (C). Proteins were separated by 2-D gel electrophoresis and stained with Coomassie blue. A segment of each 2-D gel is shown.

A proteomic analysis of LB400 cells grown in 3-HPA and 4-HPA as sole carbon source was performed in order to elucidate catabolic enzymes involved in HPAs degradation. 2-DE analysis revealed the induction of an enzyme from the homogentisate central pathway during growth of strain LB400 in 3-HPA and 4-HPA (Fig. 6). The polypeptide was identified by MALDI-TOF as fumarylacetoacetate hydrolase HmgB, which catalyzes the conversion of fumarylacetoacetate into fumarate and acetoacetate. The protein sequence possesses 91% identity with HmgB protein from *Burkholderia phytofirmans* PsJN. The induction of HmgB during growth on 3-HPA, was 3.5-fold compared to glucose-grown cells. During growth on 4-HPA, induction of this protein was 2.5-fold compared to cells grown on glucose. The corresponding *hmgB2* gene (BxeA3899) is adjacent to *hmgA2* in strain LB400 genome. This result correlates with the expression of *hmgA2* gene observed under the same growth conditions by RT-PCR. The induction of an enzyme belonging to the homogentisate ring-cleavage pathway is in accordance with the degradation of HMG and the increased HMG dioxygenase activity in strain LB400 during growth in 3-HPA and 4-HPA. Inducible expression of two enzymes from the homogentisate pathway observed during growth of strain LB400 in HPAs indicate that this catabolic pathway is involved in 3-HPA and 4-HPA degradation by strain LB400. The lower synthesis of HmgB protein in *B. xenovorans* LB400 grown in glucose indicates a basal expression of the catabolic *hmgB1* gene during growth on this carbon source.

## Discussion

**Figure 7:**
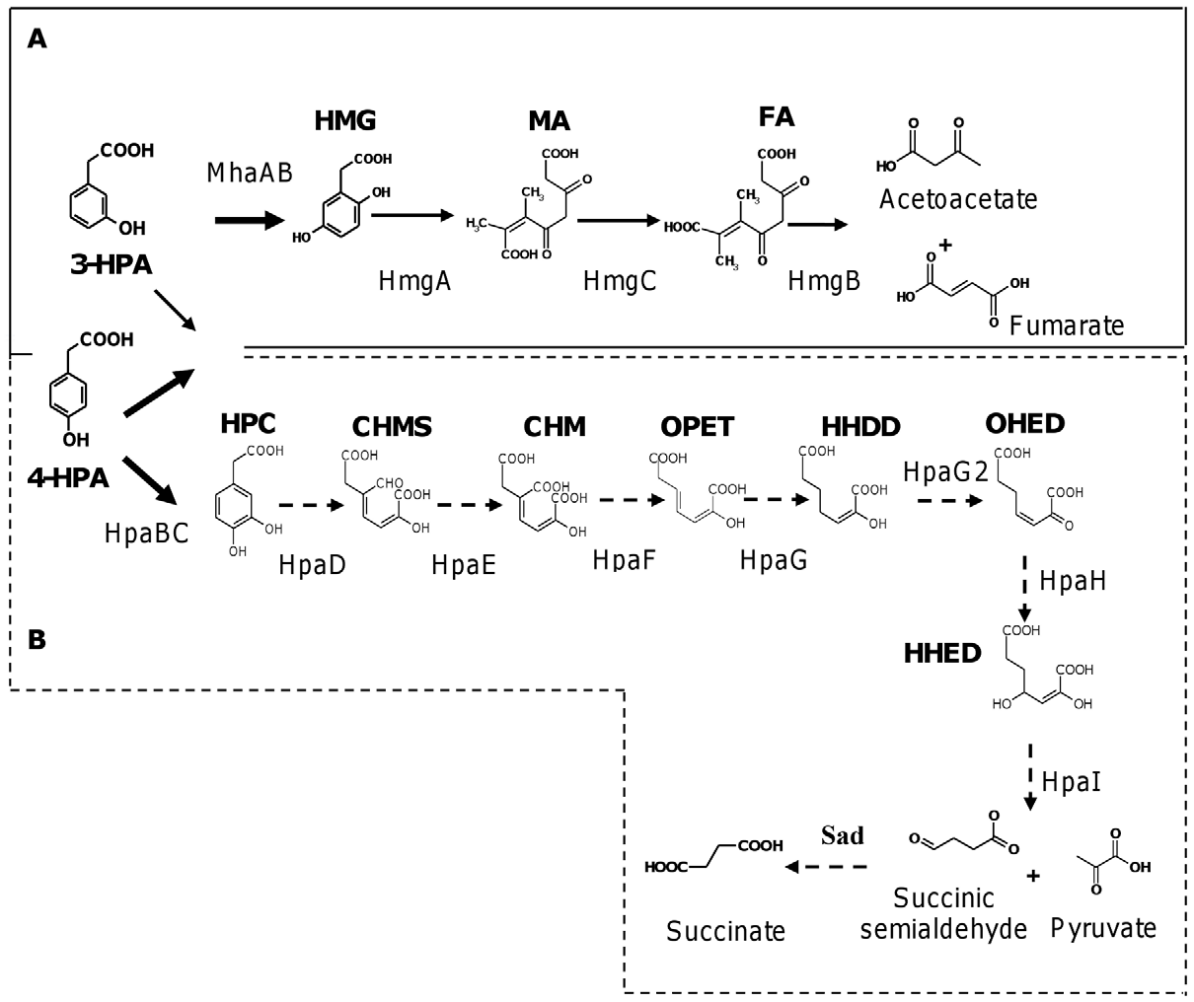
Model of 3-HPA and 4-HPA catabolic pathways in *B. xenovorans* LB400. Proposed main routes for hydroxyphenylacetates oxidation in LB400 are indicated with thick arrows, whereas the not preferentially oxidation route is indicated with a thin arrow. A, Hydroxyphenylacetates catabolism via *ortho*-cleavage pathway (continuous line). The substrates and products are: 3-HPA (3-hydroxyphenylacetate); 4-HPA (4-hydroxyphenylacetate); HMG (homogentisate); MA (maleylacetoacetate) and FA (fumarylacetoacetate). The enzymes are MhaAB (3-HPA 6-hydroxylase); HmgA (HMG 1,2-dioxygenase); HmgC (maleylacetate isomerase), HmgB (fumarylacetate hydrolase). B, Hydroxyphenylacetates catabolism via *meta*-cleavage pathway (dotted line). The metabolites are: HPC (homoprotocatechuate), CHMS (5-carboxymethyl-2-hydroxy-muconic semialdehyde), CHM (5-carboxymethyl-2-hydroxy-muconic acid), OPET (5-oxo-pent-3-ene-1,2,5-tricarboxylic acid), HHDD (2-hydroxy-hept-2,4-diene-1,7-dioic acid), OHED (2-oxo-hept-3-ene-1,7-dioic acid), and HHED (2,4-dihydroxy-hept-2-ene-1,7-dioic acid). The enzymes are HpaBC (4-HPA monooxygenase), HpaD (HPC 2,3-dioxygenase), HpaE (CHMS dehydrogenase), HpaF (CHM isomerase), HpaG (OPET decarboxylase), HpaH (OHED hydratase), HpaI (HHED aldolase), and Sad (succinic semialdehyde dehydrogenase). We propose that in strain LB400 3-HPA is preferentially channeled into the homogentisate central pathway, whereas 4-HPA is channeled actively in both homogentisate and homoprotocatechuate central pathways.

This study has shown that the homogentisate and the homoprotocatechuate central pathways are involved in 3-HPA and 4-HPA catabolism by *B. xenovorans* strain LB400. 3-HPA and 4-HPA isomers are used by *B. xenovorans* LB400 as sole carbon and energy source for growth, indicating active peripheral and central catabolic pathways. Bacterial catabolism of hydroxyphenylacetates, particularly 3-HPA and 4-HPA compounds have been described to channel either the homogentisate or the homoprotocatechuate central pathways. Interestingly, this report showed that LB400 growth on 3-HPA and 4-HPA separately funnels into both central pathways. Similarly, in *P. putida* strain U, different catabolic pathways were reported for 4-HPA and 3-HPA degradation. It is worth noting that strain LB400 showed a faster growth in 3-HPA (5 mM) than *P. putida* strain U in 3-HPA (10 mM) [30]. In this study, strain LB400 reached high biomass using 4-HPA as sole carbon source (turbidity_525 nm_ of 1.1) (data not shown). In contrast, in similar media with 4-HPA as carbon source *P. putida* U attains lower biomass (turbidity_540 nm_ of 0.3) [27]. The MhaAB protein from strain U is highly specific for 3-HPA substrate to yield HMG [30], which is further degraded by the *hmgABC* gene products [19], [30]. Interestingly, upstream to the *hmgA1B1C1* cluster in the LB400 genome, two ORFs (BxeA2727 and BxeA2726) with high identity to the *mhaAB* genes from strain U were found. The MhaA flavoprotein component (566 aa) and the MhaB coupling protein (60 aa) possess a similar length to the aminoacid sequence deduced from BxeA2727 (560 aa) and BxeA2726 (60 aa) genes of strain LB400. In a previous study, the BxeA2727 was annotated as a putative 3-(3-hydroxy-phenyl)propionate hydroxylase (MhpA) [3]. We propose that BxeA2727 and BxeA2726 of strain LB400 encode the two components of the hydroxylating enzyme MhaAB that is involved in catabolism of 3-HPA via HMG (Fig. 7A). However, further analyses are required to confirm the involvement of the *mhaAB* genes in 3-HPA degradation by strain LB400.

Alternatively, 4-HPA degradation in *P. putida* strain U is mediated by a 4-HPA 3-hydroxylase yielding HPC [27]. Interestingly, the results presented above indicate that both 4-HPA and 3-HPA compounds are degraded via HPC in strain LB400. However, based on *hmgA* and *hpaD* gene expression analyses and HMG and HPC dioxygenase activities, we postulate that 3-HPA in strain LB400 is preferentially channelled into the homogentisate central pathway. A previous report of 4-HPA catabolism showed that 4-HPA 3-hydroxylase from *E. coli* strain W hydroxylates 4-HPA and also 3-HPA yielding HPC [29]. A search of *hpaB* and *hpaC* genes in LB400 genome showed a gene (BxeB2309) with high identity to the *hpaC* gene encoding the 4-HPA 3-hydroxylase coupling protein of *E. coli* strain W located 309 b distant from the *hpaGEDFHI* gene cluster in strain LB400. The adjacent gene (BxeB2308) probably encodes a FAD-dependent monooxygenase distantly related to the HpaB hydroxylase component from *E. coli* W [29]. We propose that the BxeB2308 and BxeB2309 genes encode the two components of a hydroxylase involved in 3-HPA and 4-HPA catabolism (Fig. 7B).

Additionally, this study demonstrated that both chromosomally encoded homogentisate and homoprotocatechuate central pathways are functional in strain LB400. Resting cell assays indicated that strain LB400 degraded HMG and HPC compounds. Moreover, a high HMG dioxygenase activity was measured in 3-HPA and 4-HPA-grown cells (82 and 75 mU/mg of protein, respectively), whereas no activity was detected in glucose-grown cells (Fig. 4A). A similar HMG dioxygenase activity (68 mU/mg of protein) was previously reported in crude extracts of *P. putida* U cells grown on tyrosine or phenylalanine [19]. LB400 cells grown on 3-HPA and 4-HPA showed high HPC dioxygenase activities, whereas a lower activity was measured in glucose-grown cells (Fig. 4B). These results indicate that: i) key ring-cleavage dioxygenases of the homogentisate and the homoprotocatechuate pathways are functional, and ii) the homogentisate and the homoprotocatechuate central pathways are induced in strain LB400 during growth on 3-HPA and 4-HPA.

During growth of strain LB400 on 3-HPA, expression of the *hmgA1* gene (BxeA2725) and *hmgA2* gene (BxeA3900) were observed by RT-PCR. The *hmgA1* gene is part of the *hmgABC* gene cluster in LB400, whereas the *hmgA2* gene is clustered only with *hmgB2* gene. In contrast, during 4-HPA degradation, the expression of *hmgA2* gene but not of *hmgA1* gene was observed. The results indicate that: i) the *hmgA1B1C1* cluster of strain LB400 is up regulated during 3-HPA metabolism but not during 4-HPA metabolism and, ii), two *hmgA* gene copies are transcribed during growth on 3-HPA. It is worth noting that these two genes have not identical DNA sequence. It is likely that one of the *hmgA* genes was acquired via horizontal gene transfer, which has been an important source of genes in strain LB400 [8]. These data indicates that growth on 3-HPA and 4-HPA are mediated by different cellular enzymatic components. During degradation of 3-HPA, it is likely that two *hmgA* gene copies are used, suggesting that enhanced catabolic abilities are required. Probably, the homogentisate pathway in strain LB400 is regulated at the level of HMG dioxygenase to prevent accumulation of catabolic intermediates that may have toxic effects on the cell. Expression of two gene copies encoding key enzymes may enhance catabolic machinery of strain LB400. The presence of multiples *hmg* gene copies spread in LB400 genome probably enhance its catabolic capabilities. The *hmgAB* arrangement, not linked with *hmgC* gene has also been observed in *Silicibacter pomeroyi*, *Pseudomonas syringae*, *Ralstonia solanacearum*, *Bordetella bronchiseptica* and *Cupriavidus necator* [7], [19]. The use of the homogentisate pathway encoded by the *hmgAB* gene cluster has also proposed during growth on 3-HPA and 4-HPA in *C. necator* strain JMP134; in this strain the homoprotocatechuate pathway is absent [7]. The presence of multiples genes copies encoding chlorocatechol 1,2-dioxygenase in *C. necator* JMP134 are required for efficient chlorocatechol degradation during growth of strain JMP134 on 3-chlorobenzoate [38]. Enhanced degradation avoids accumulation of toxic metabolic intermediates such as catechols [5], [39]. The toxicity of aromatic compounds and their metabolic intermediates for strain LB400 has been reported [9], [31], [35].

In addition to the expression of *hmgA* genes, the *hpaD* gene encoding HPC dioxygenase was expressed in LB400 during growth on 3-HPA and 4-HPA. A lower *hpaD* expression on glucose was observed. This in accordance with high HPC dioxygenase observed during growth in 3-HPA and 4-HPA and a lower activity observed in glucose-grown cells. In *Gammaproteobacteria*, 4-HPA is commonly degraded through the homoprotocatechuate pathway. 4-HPA degradation via HPC has been studied in some *E. coli* strains such as strain W that lacks the homogentisate pathway [2], [18], [29]. As mentioned above, the 4-HPA hydroxylase from *E. coli* strain W is able to hydroxylate a wide range of substrates, including 3-HPA [29]. Thus, we propose that the homoprotocatechuate pathway is an additional convergent route involved in 3-HPA and 4-HPA degradation in *B. xenovorans* LB400.

Proteomic analyses of LB400 cells grown on 3-HPA and 4-HPA provided additional evidence that the homogentisate central pathway is involved in the catabolism of 3-HPA and 4-HPA. The induction of fumarylacetoacetate hydrolase HmgB by 3-HPA and 4-HPA was observed. The HmgB protein was induced in 3-HPA-grown cells (3.5-fold) and in 4-HPA-grown cells (2.5-fold) compared to glucose-grown cells. As suggested above, 3-HPA hydroxylation mediated by the *mhaAB* gene products from strain LB400 is in accordance with 3-HPA catabolism via HMG revealed by proteomic analyses. 4-HPA degradation also has been reported to funnel into the homogentisate route, by hydroxylation at carbon 1 in *Pseudomonas acidovorans* and *Azoarcus evansii* [40], [41]. Therefore, it is likely that 4-HPA is metabolized through the homogentisate pathway in LB400, in accordance with HmgB2 induction during 4-HPA metabolism observed by 2D-PAGE. The *hmgB2* gene is adjacent to the *hmgA2* gene (BxeA3900) in C1 of strain LB400. This is in accordance with *hmgA2* expression observed during growth in 3-HPA and 4-HPA. The induction of HmgB protein suggests an up-regulation of the homogentisate pathway during 3-HPA and 4-HPA degradation in strain LB400. Moreover, HMG dioxygenase activity measured in crude extracts of strain LB400 suggested also induction of *hmg* catabolic genes. The HmgR regulator protein from *P. putida* strain U is an IclR-type regulator divergently transcribed from *hmgABC* cluster and acts as a repressor of *hmg* genes [19]. However, a gene similar to the HmgR protein from *P. putida* U was not identified in the *hmgA1B1C1* cluster neighborhood and other regions of the genome of strain LB400. The transcription analyses suggested that expression of the homogentisate and homoprotocatechuate pathways are controlled at transcriptional level in LB400, in which the presence of specific substrates such as 3-HPA and 4-HPA are required. A gene encoding a HpaR regulator is located adjacent to the *hpaGEDFHI* gene cluster in LB400 genome. It has been determined that the HpaR protein of *E. coli* strain C regulates transcription of the *hpaGEDFHI* cluster and it is induced by 4-HPA or HPC [26]. It is likely that 4-HPA, 3-HPA or metabolic intermediates such as HPC may regulate expression of *hpa* cluster in LB400 mediated by HpaR (Fig. 1B).

Genome characterization of *B. xenovorans* LB400 revealed interesting catabolic capabilities. In this work, we provided evidence on the functionality of the two chromosomally encoded homogentisate and homoprotocatechuate central pathways. In addition, it was shown that the 3-HPA and 4-HPA catabolic peripheral pathways are active. Moreover, two functional *hmgA* genes were used by LB400 during 3-HPA catabolism. Based on growth and degradation assays, gene expression analyses, protein synthesis and key dioxygenases activities, we postulate that in strain LB400 3-HPA and 4-HPA separately funnel into both homogentisate and homoprotocatechuate central pathways. Probably, 3-HPA is preferentially channelled into the homogentisate central pathway, whereas 4-HPA is channelled actively in both central pathways. This study reveals the wide aromatic catabolic repertoire of strain LB400, linking the abilities predicted *in silico* with those observed at functional level. Furthermore, the utilization of both *ortho*- and *meta*-cleavage pathways in the degradation of two isomers reflects the amazing metabolic plasticity of this bacterium.
